# Divergence of functional effects among bacterial sRNA paralogs

**DOI:** 10.1186/s12862-017-1037-5

**Published:** 2017-08-22

**Authors:** I-Chen Kimberly Chen, Gregory J. Velicer, Yuen-Tsu Nicco Yu

**Affiliations:** 1 Department of Biology, Indiana University, Bloomington, IN47405 USA; 2 Institute of Integrative Biology, ETH Zurich, CH-8092 Zurich, Switzerland; 3 Present address: School of Biological Sciences, Georgia Institute of Technology, Atlanta, GA30332 USA

**Keywords:** Bacterial development, Gene duplication, Multicellularity, Myxobacteria, Small non-coding RNAs, Regulation of gene expression

## Abstract

**Background:**

Non-coding small RNAs (sRNAs) regulate a variety of important biological processes across all life domains, including bacteria. However, little is known about the functional evolution of sRNAs in bacteria, which might occur via changes in sRNA structure and/or stability or changes in interactions between sRNAs and their associated regulatory networks, including target mRNAs. The sRNA Pxr functions as a developmental gatekeeper in the model cooperative bacterium *Myxococcus xanthus*. Specifically, Pxr prevents the initiation of fruiting body development when nutrients are abundant. Previous work has shown that Pxr appears to have a recent origin within a sub-clade of the myxobacteria, which allowed us to infer the most recent common ancestor of *pxr* and examine the divergence of Pxr since its origin*.*

**Results:**

To test for inter-specific divergence in functional effects, extant *pxr* homologs from several species and their inferred ancestor were introduced into an *M. xanthus* deletion mutant lacking *pxr*. Both the inferred ancestral *pxr* and all extant alleles from species containing only one copy of *pxr* were found to control development in *M. xanthus* in a qualitatively similar manner to the native *M. xanthus* allele. However, multiple paralogs present in *Cystobacter* species exhibited divergent effects, with two paralogs controlling *M. xanthus* development but two others failing to do so. These differences may have occurred through changes in gene expression caused by apparent structural differences in the sRNA variants encoded by these paralogs.

**Conclusions:**

Taken together, our results suggest that Pxr plays a common fundamental role in developmental gene regulation across diverse species of myxobacteria but also that the functional effects of some Pxr variants may be evolving in some lineages.

**Electronic supplementary material:**

The online version of this article (doi:10.1186/s12862-017-1037-5) contains supplementary material, which is available to authorized users.

## Background

Non-coding small RNAs are prevalent in regulating gene expression across all domains of life, from eukaryotes, archaea to bacteria [1–6] and include *trans*-encoded microRNAs (miRNAs) in animals and plants and small regulatory RNAs (sRNAs) in bacteria. These small RNAs modulate gene expression at the post-transcriptional level by base-pairing with their mRNA targets, resulting in mRNA degradation, inhibition of translation, or both. In bacteria, these small regulators play important roles in a variety of physiological processes, including stress responses [7–11], outer membrane protein synthesis [12–15], virulence [16] and social behaviors [7, 17, 18].

Bacterial sRNAs are often ~100 nucleotides in length (but can also be larger, e.g. [19, 20]), transcribed from the intergenic regions in bacterial genomes with their own promoters and commonly have multiple mRNA targets [2, 3]. They are predicted to fold into stable stem-loop structures including a ρ-independent terminator at the 3′ end. The function of an sRNA is determined by at least a seed region of ~6–8 bases that are complementary to a region of their mRNA targets, and this region is one of the most conserved features of homologous sRNA sequences [21, 22]. Even a single nucleotide change in this region can abolish sRNA-based regulation [7, 23–25], suggesting strong selective constraints on this region. Nevertheless, other sequence regions might also be prominent for function [23, 26].

Several proteins are known to participate in sRNA gene regulation. For example, many characterized sRNAs in enteric bacteria require the RNA chaperone Hfq for interactions with mRNA targets [27], although a number of sRNAs in other species require proteins other than Hfq [28, 29]. Ribonucleases also contribute to sRNA activities and levels. Most base-pairing sRNAs are transcribed as independent units, but few are processed in some manner [7, 30–32]. RNases E and RNase III also facilitate target mRNA degradation [33–35].

Recent studies have shown that the phylogenetic distributions and sequences of sRNAs tend to be lineage-specific [21, 36–39], suggesting independent origins of sRNAs in specific lineages followed by phylogenetically limited patterns of duplication, diversification and loss rather than a high rate lateral sRNA gene transfer across large phylogenetic distances [40]. For instance, a large fraction of *Escherichia coli* sRNAs has accumulated in the Enterobacteriales order since it split from the rest of the γ-proteobacteria [36].

Nevertheless, the functional evolution of sRNAs and how they coevolve with their associated regulatory networks remain poorly understood in most systems. In enteric bacteria, the SgrS sRNAs associated with glucose-phosphate stress appear to have coevolved with their associated networks, as many *sgrS* homologs from different species fail to control the same target mRNAs in *E. coli* that the respective *E. coli-*specific allele regulates [41]. However, *sgrS* is unique in that it also encodes a protein-coding gene *sgrT*, which regulates glucose-phosphate stress via an unknown mechanism [42]. It is thus unclear how representative *sgrS* may be of other sRNA systems with respect to evolutionary questions.

In this study, we sought to investigate evolution of an sRNA - Pxr - that controls fruiting body development in the myxobacteria. Many species of myxobacteria (δ-proteobacteria, order Myxococcales), including *Myxococcus xanthus*, survive starvation by multicellular development into fruiting bodies that yield stress-resistant spores [43]. In *M. xanthus*, Pxr functions as a developmental gatekeeper that prevents the initiation of fruiting body development when nutrients are abundant [7]. The *pxr* gene is located downstream of the σ^54^-dependent response regulator *nla19*, appears to be transcribed from an upstream σ^54^ promoter and is predicted to fold into a stable triple-stem-loop structure [7]. There are two *pxr*-specific sRNA forms, Pxr-L (long) and Pxr-S (short), and Pxr-S is thought to be the product of Pxr-L processing [7]. Both Pxr-L and Pxr-S are expressed at high levels during vegetative growth, but Pxr-S appears to be the primary negative regulator because it (but not Pxr-L) is rapidly and greatly diminished upon starvation, which presumably allows development to proceed [7]. Pxr appears to have a recent evolutionary origin in the lineage basal to the suborder Cystobacterineae within the Myxococcocales order [38]. Most species within this suborder contain a single copy of *pxr*; however, tandem paralogs were found within species of the genus *Cystobacter* [38].

Pxr was discovered from an evolution experiment in which a developmentally defective *M. xanthus* strain (‘OC’, obligate cheater) evolved into a strain with restored developmental proficiency (‘PX’, phoenix) due to a mutation that deactivated Pxr [44]. OC is a descendant of the developmentally proficient wild-type strain GJV1 and differs from GJV1 by 14 mutations that accumulated during laboratory evolution in a nutrient-rich liquid environment [45, 46]. This cheater strain is defective at development in clonal groups but can exploit GJV1 in chimeric groups to sporulate more efficiently than GJV1. Pxr-S, the primary regulatory form of Pxr, is constitutively expressed in OC, which in turn down-regulates development even when nutrients are depleted. Accordingly, OC is defective at fruiting body formation and sporulation on nutrient-free plates. Strain PX emerged from OC after a cheater-induced population crash [44]. PX differs from OC by a single C→A mutation in the first loop of Pxr, which abolishes the regulatory function of Pxr and confers the phenotype of fruiting body formation and high spore production even though the defective variant of Pxr-S continues to be produced [7, 44]. Taken together, these results suggested that Pxr is a negative regulator of *M. xanthus* development that blocks the initiation of fruiting body formation during vegetative growth. Indeed, deletion of *pxr* from the wild-type strain GJV1 allows development to proceed at high nutrient levels [7].

The fact that OC constitutively expresses Pxr-S provides an excellent opportunity to examine the functionality of *pxr* variants in its genetic background. Homologs of *pxr* proficient at blocking development in the OC background will demonstrate very low spore production, and vice versa. In this study, we first reconstructed the previously-inferred most recent common ancestor of extant *pxr* homologs and introduced it into an OC deletion mutant lacking *pxr* (hereafter “OC Δ*pxr*”, which exhibits high sporulation like strain PX [44]) to test whether the *pxr* ancestral allele can restore the regulatory function of Pxr observed in *M. xanthus*. Next, we introduced *pxr* homologs from species with only one copy of this gene and other species with multiple *pxr* paralogs to test for functional divergence of the Pxr regulatory system in the myxobacteria.

## Results

**Fig. 1:**
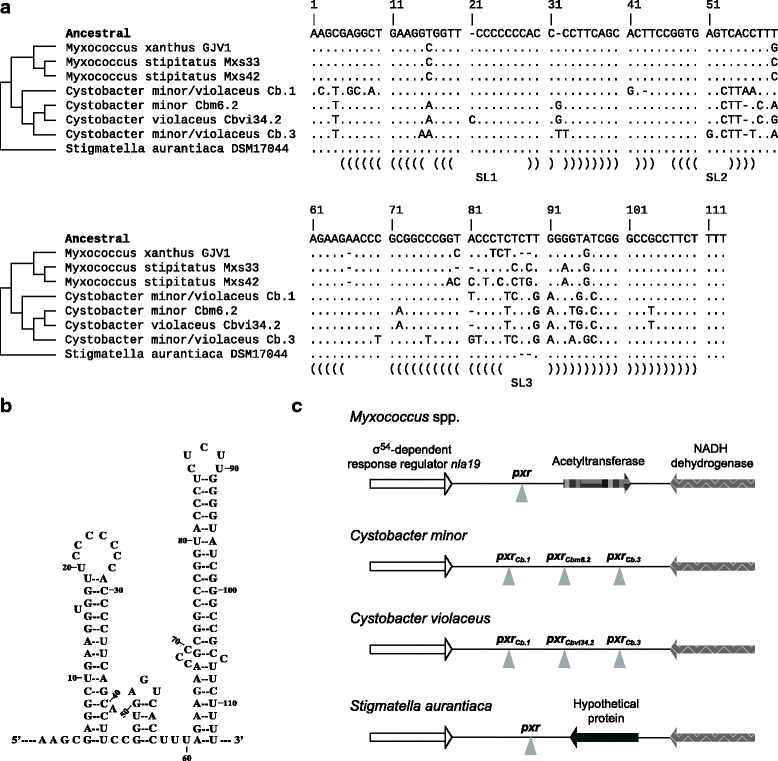
**a** Nucleotide sequences of the inferred *pxr* ancestor and extant homologs at every site from 5′ to 3′. A *dot* indicates a nucleotide identical to the corresponding site for the inferred ancestor at the internal node shared by the non-*Stigmatella* homologs, whereas *letters* show nucleotide differences. The cladogram of the *pxr* homologs is shown on the left. The consensus stem-loops based on the single-copy homologs are annotated as SL1, SL2 and SL3 at the bottom. *Brackets* represent nucleotides positioned on the stems. **b** The predicted secondary structure of the Pxr ancestor, which has a calculated self-folding free energy of −57.5 kcal/mol. The nucleotide numbers correspond to the alignment positions in (**a**). **c** Genetic organization of *pxr* alleles and their neighboring genes in different myxobacterial species. Gene and intergenic region lengths are not drawn to scale

**Fig. 2:**
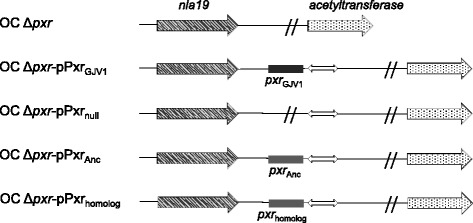
Genetic organization of the strain OC Δ*pxr* and the constructed strains in this study. The *double-arrowed blocks* are integrated pCR2.1 vector fragments and the symbol // indicates the deletion of *pxr*

We first describe results with the inferred *pxr* ancestral allele and then results with extant homologs from different species to examine the functional divergence of Pxr since its origin in the myxobacteria. Nucleotide sequences of the inferred ancestor and extant homologs at every site are shown in Fig. 1a. As described in the Materials and Methods, for each constructed strain that carries a unique allele of *pxr*, we examined two or three independently isolated clones for their developmental phenotypes. Because no differences among independent clones of identical constructs were detected either within or among replicates, we used within-replicate means across independent clones as data points. For clarity, for the three control strains GJV1, OC and OC Δ*pxr*, we retain their original designations. For the two isogenic control constructs that differ only in the presence or absence of the *M. xanthus* GJV1 *pxr* allele, we refer to them as “*pxr*_*GJV1*_” and “*pxr*_*null*_”. For constructs with different versions of *pxr*, we label them according to their strains of origin (e.g. “*pxr*_*Mxs33*_” for the *pxr* homolog from *M. stipitatus* strain Mxs33) or as “*pxr*_*Anc*_” for the inferred﻿ ancestral allele. Experimental strains of *M. xanthus *new to this study were derived from OC Δ*pxr* and were generated by homologous integration of plasmids that carry the 429-nt region preceding *pxr* in *M. xanthus* in conjunction with different versions of *pxr*. A list of strains used is provided in Table 1 and the *pxr* mero-dioploid region of each constructed strain is shown on Fig. 2.

**Table 1:** List of *M. xanthus* strains and plasmids used in this study

Strain	Genotype	*pxr* allele description	Reference/source
GJV1	Derivative isolate of *Myxococcus xanthus* DK1622	*M. xanthus* GJV1 *pxr*	[44, 47]
GVB207.3	Evolutionary descendant of GJV1 (herein “OC”)	*M. xanthus* GJV1 *pxr*	[44, 46]
GJV207	GVB207.3 Δ*pxr* (herein “OC Δ*pxr*”)	In-frame deletion of *pxr*	[7]
NY01	GJV207 *intergenic*::pPxr_GJV1_, kan^R^	*M. xanthus* GJV1 *pxr*	This study
NY02	GJV207 *intergenic*::pPxr_null_, kan^R^	Intergenic region preceding *pxr* only	This study
KC00	GJV207 *intergenic*::pPxr_Anc_, kan^R^	The inferred *pxr* ancestor shared by *Myxococcus* and *Cystobacter* spp.	This study
KC01	GJV207 *intergenic*::pPxr_Mxs33_, kan^R^	*Myxococcus stipitatus* Mxs 33 *pxr*	This study
KC02	GJV207 *intergenic*::pPxr_Mxs42_, kan^R^	*Myxococcus stipitatus* Mxs 42 *pxr*	This study
KC03	GJV207 *intergenic*::pPxr_*Cb.1*_, kan^R^	*pxr*_Cb.1_ in both *Cystobacter minor* Cbm 6 and *Cystobacter violaceus* Cbvi 34	This study
KC04	GJV207 *intergenic*::pPxr_*Cbm6.2*_, kan^R^	*pxr*_Cbm6.2_ in *C. minor* Cbm 6	This study
KC05	GJV207 *intergenic*::pPxr_*Cbvi34.2*_, kan^R^	*pxr*_Cbvi34.2_ in *C. violaceus* Cbvi 34	This study
KC06	GJV207 *intergenic*::pPxr_*Cb.3*_, kan^R^	*pxr*_Cb.3_ in both *C. minor* Cbm 6 and *C. violaceus* Cbvi 34	This study
KC07	GJV207 *intergenic*::pPxr_*Sga*_, kan^R^	*Stigmatella aurantiaca* DSM 17044 *pxr*	This study
Plasmid	Description		Reference/source
pCR2.1	Cloning vector		Invitrogen
pPxr_GJV1_	pCR2.1 with a 537-nt fragment that contains the 429-nt intergenic fragment and the 108-nt *pxr* coding region in *M. xanthus*		This study
pPxr_null_	pCR2.1 with only the 429-nt intergenic fragment in *M. xanthus*		This study
pPxr_Anc_	pCR2.1 with the 429-nt intergenic fragment in conjunction with the inferred *pxr* ancestor shared by *Myxococcus* and *Cystobacter*. spp.		This study
pPxr_Mxs33_	pCR2.1 with the 429-nt intergenic fragment in conjunction with the *pxr* allele from *M. stipitatus* strain Mxs 33		This study
pPxr_Mxs42_	pCR2.1 with the 429-nt intergenic fragment in conjunction with the *pxr* allele from *M. stipitatus* strain Mxs 42		This study
pPxr_Cb.1_	pCR2.1 with the 429-nt intergenic fragment in conjunction with *pxr*_Cb.1_ in both *C. minor* Cbm 6 and *C. violaceus* Cbvi 34		This study
pPxr_Cbm6.2_	pCR2.1 with the 429-nt intergenic fragment in conjunction with *pxr*_Cbm6.2_ in *C. minor* strain Cbm 6		This study
pPxr_Cbvi34.2_	pCR2.1 with the 429-nt intergenic fragment in conjunction with *pxr*_Cbvi34.2_ in *C. violaceus* strain Cbvi 34		This study
pPxr_Cb.3_	pCR2.1 with the 429-nt intergenic fragment in conjunction with *pxr*_Cb.3_ in both *C. minor* Cbm 6 and *C. violaceus* Cbvi 34		This study
pPxr_Sga_	pCR2.1 with the 429-nt intergenic fragment in conjunction with the *pxr* allele from *S. aurantiaca* strain DSM 17044		This study

“*intergenic*” refers to a 429-nt fragment that contains the 3′-terminal 167-nt of *nla19* and the 262-nt intergenic region between *nla19* and *pxr* in *M. xanthus*

### The inferred ancestral *pxr* allele negatively regulates development

The sequence of *pxr* ancestral to homologs within the *Myxococcus* and *Cystobacter* genera was previously inferred using the homolog from the distantly related genus *Stigmatella* as an outgroup (all three genera belong to the suborder Cystobacterineae in the order Myxococcocales) [38] (Fig. 1b). Although we refer to this allele as ancestral, it is not necessarily ancestral to the *Stigmatella* homolog and differs from it only by the presence of two bases (CU) in the third loop that are absent in the *Stigmatella* sequence. Between the inferred *pxr* ancestor and the extant *M. xanthus* GJV1 homolog there are seven base differences. Six of these differences are either near or on the third and last stem-loop of Pxr. Among them three are single base substitutions: a U→G change in the single-stranded region between the second and third stem-loops and a pair of complementary changes on the third stem (U→C combined with A→G). The other three differences are single base deletions: one at the base of the bulge on the left side of the third stem, one nearby the third loop and one in the loop. The seventh difference is a single base change U→C at the bulge on the left side of the first stem.

**Fig. 3:**
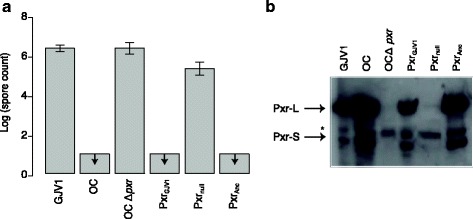
**a** The ancestral Pxr blocks *M. xanthus* development in a DK1622/GJV1-derived genomic background. Spore production of the OC Δ*pxr* strain carrying the *pxr* ancestor and the control strains are shown. *Arrows* indicate that no spores were observed at the lower limit of detection. Error bars represent standard deviations. **b** Northern blot showing the expression of Pxr-L and Pxr-S produced by the OC Δ *pxr* strain carrying the *pxr* ancestor and the control strains. The *asterisk* marks binding of the probe to non-Pxr RNAs

We examined the proficiency of the Pxr ancestor at controlling development in *M. xanthus* by measuring spore production on nutrient-free agar plates. Pxr_Anc_ blocked development just as effectively as Pxr_GJV1_, as no spores at the lower limit of detection were obtained from the OC Δ*pxr*-derived strain carrying either allele (Fig. 3a). This result indicates that the seven base differences between the ancestor and the *M. xanthus* copy do not affect the developmental phenotype assayed here. The Northern blot showed that *pxr*_*Anc*_ was expressed into both Pxr-L and Pxr-S at similar levels and sizes as the extant *M. xanthus* allele (Fig. 3b).

### *pxr* paralogs diverged greatly in sequence and functional effects

Seven previously identified *pxr* homologs were examined in this study. Three of them from phylogenetically diverse species (two from *M. stipitatus* strains Mxs33 and Mxs42 and one from *Stigmatella aurantiaca* DSM17044) are single-copy orthologs with a degree of sequence conservation similar to that of the 23S rRNA gene [38]. Four of them are paralogs found in *Cystobacter minor* Cbm 6 and *C. violaceus* Cbvi 34. Both of these strains carry multiple *pxr* paralogs in tandem (*pxr*_*Cb.1*_ in both strains, *pxr*_*Cbm6.2*_ in *C. minor, pxr*_*Cbvi34.2*_ in *C. violaceus* and *pxr*_*Cb.3*_ in both strains from 5′ to 3′) (Fig. 1c). The divergence among these paralogs within each *Cystobacter* species is greater than their orthologous divergence across species. For instance, the estimated evolutionary distances between *pxr*_*Cb.1*_ vs. *pxr*_*Cbm6.2*_, *pxr*_*Cbm6.2*_ vs. *pxr*_*Cb.3*_, and *pxr*_*Cb.1*_ vs. *pxr*_*Cb.3*_ in *C. minor* Cbm 6 are 14.4%, 17.4% and 22.6% (Table 2), but *pxr*_*Cb.1*_ and *pxr*_*Cb.3*_ in *C. minor* Cbm 6 and *C. violaceus* Cbvi 34 are identical to each other and *pxr*_*Cbm6.2*_ *and pxr*_*Cbvi34.2*_ only differ by two nucleotide positions across these two species. We thus infer that these paralogs originated by gene duplication in a common ancestor of *C. minor* and *C. violaceus* and diverged greatly from each other prior to the split between these species and that subsequent inter-specific divergence occurred only in the second paralogs.

**Table 2:** Estimates of evolutionary distances between each pair of *pxr* homologs

	GJV1	Mxs33	Mxs42	Cb.1	Cbm6.2	Cbvi34.2	Cb.3	Sga
GJV1								
Mxs33	0.064							
Mxs42	0.085	0.051						
Cb.1	0.322	0.285	0.421					
Cbm6.2	0.233	0.246	0.351	0.144				
Cbvi34.2	0.219	0.246	0.351	0.145	0.009			
Cb.3	0.344	0.282	0.417	0.226	0.174	0.189		
Sga	0.076	0.052	0.113	0.219	0.179	0.179	0.298	

The distances were determined using the maximum composite likelihood model implemented in MEGA v. 5.0 [67] and the numbers shown are base substitutions per site between sequences

All *pxr* homologs examined are located in the intergenic region between the σ^54^-dependent response regulator *nla19* and a predicted NADH dehydrogenase gene (predicted genes *Mxan_1078* and *Mxan_1080* in the *M. xanthus* genome; [47]) but other neighboring gene content has diversified among genera (Fig. 1c). These homologs also appear to be transcribed from an upstream σ^54^ promoter like the one in *M. xanthus* and are predicted to fold into stable multi-stem-loop structures [38]. The two extant *Myxococcus* alleles, the *Stigmatella* allele and the second *Cystobacter* paralogs in both *C. minor* and *C. violaceus* (Pxr_Cbm6.2_ and Pxr_Cbvi34.2_) examined here are predicted to form three stem-loops, but the first and third *Cystobacter* paralogs (Pxr_Cb.1_ and Pxr_Cb.3_) are predicted to fold into only two long stem-loops and lack the second short stem-loop predicted for the other alleles [38]. The consensus secondary structure of Pxr based on the alignment of single-copy orthologs is indicated in Fig. 1a.

**Fig. 4:**
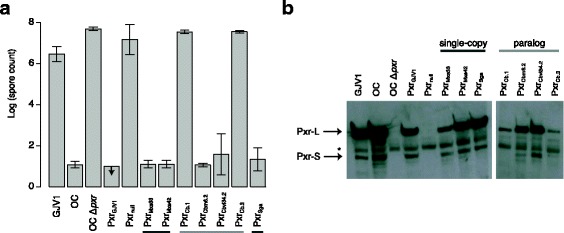
**a** Single-copy Pxr homologs from different species blocked *M. xanthus* development but there is large functional variation among the paralogs in *Cystobacter*. Spore production by OC Δ*pxr*-derived strains carrying integrated *pxr* homologs from different myxobacterial species are shown. *Dark grey* bars indicate single-copy Pxr homologs and *light grey bars* indicate Pxr paralogs. *Arrows* indicate that no spores were produced at the lower limit of detection. Error bars represent standard deviations. **b** Northern blot showing the expression of Pxr-L and Pxr-S produced by the OC Δ*pxr*-derived strains integrated with *pxr* homologs from different species of myxobacteria and the control strains. The *asterisk* marks binding of the probe to non-Pxr RNAs

To ensure all *pxr* homologs are transcribed in the same manner as the native *M. xanthus pxr* allele, all the homologs in our constructs are preceded by the 429-nt region upstream of the *pxr* gene in *M. xanthus* (this region contains the 3′-terminal 167-nt of *nla19* and the 262-nt intergenic region between *nla19* and *pxr*)*.* Despite extensive efforts to clone completely accurate sequences of *pxr* homologs, cloned sequences all lacked the first adenine. Because this adenine was absent from all cloned homologs we proceeded with these sequences for functional analysis. The developmental assays showed that the *Myxococcus* and *Stigmatella* orthologs (Pxr_Mxs33_, Pxr_Mxs42_ and Pxr_Sga_) all effectively blocked *M. xanthus* development, as the OC Δ*pxr*-derived strains carrying them all produced almost no spores (Fig. 4a). These results demonstrate that nucleotides different between the orthologs tested are not essential for Pxr function and lacking the first adenine does not prevent effective blockage of development by Pxr.

In contrast, the Pxr paralogs in *Cystobacter* (Fig. 4a) varied greatly in their functional effects. Although both Pxr_Cbm6.2_ and Pxr_Cbvi34.2_ from *C. minor* Cbm 6 and *C. violaceus* Cbvi 34 effectively blocked spore production, Pxr_Cb.1_ and Pxr_Cb.3_ failed to do so. Spore production by the OC Δ*pxr*-derived strains carrying these two paralogs was similar to that of OC Δ*pxr* and the isogenic construct lacking any *pxr* sequence.

### Structural differences in *pxr* paralogs affect levels of Pxr accumulation in *M. xanthus*

We used Northern analysis to examine Pxr-production patterns and levels for each *pxr* homolog (Fig. 4b). The three single-copy *pxr* homologs and *pxr*_*Cbm6.2*_ and* pxr*_*Cbvi34.2*_ from *C. minor* Cbm 6 and *C. violaceus* Cbvi 34 were all expressed into both Pxr-L and Pxr-S at sizes and levels similar to the native *M. xanthus pxr* allele, supporting the developmental phenotypes we observed from the strains carrying them. For the *pxr*_*Cb.1*_ and *pxr*_*Cb.3*_ paralogs that did not block development, Pxr-L was produced at a similar size as that from the *M. xanthus* allele but in much lower amounts. Pxr-S was not detected at all from the strain carrying *pxr*_*Cb.1*_ and was only barely detected in the strain carrying *pxr*_*Cb.3*_. These results indicate that lack of Pxr-S is associated with the inability of Pxr_Cb.1_ and Pxr_Cb.3_ to block development. This lack of Pxr-S may stem from the reduced amounts of Pxr-L, which in turn might be due to the instability of RNA transcripts.

If Pxr_Cb.1_ and Pxr_Cb.3_ transcripts are less stable than those of other alleles (at least when expressed in *M. xanthus*), such instability might be caused by the sequences *per se* or structural differences caused by their sequences. At the sequence level, the nucleotide changes that occurred in both *pxr*_Cb.1_ and *pxr*_Cb.3_ but not in *pxr*_*Cbm6.2*_ and *pxr*_*Cbvi34.2*_ are at positions 57, 81 and 87 (Fig. 1a). Although the nucleotide changes at positions 57 and 81 are different in *pxr*_Cb.1_ and *pxr*_Cb.3_, changes at these two positions might affect the stability of Pxr. The change at position 87 (T→C) is shared with *pxr*_*Mxs33*_ and *pxr*_*Mxs42*_, although it is possible that the interactions between this change and the ones at 57 and 81 also affect stability. The rest of the nucleotide differences in *pxr*_Cb.1_ and *pxr*_Cb.3_ not shared with *pxr*_*Cbm6.2*_ and *pxr*_*Cbvi34.2*_ do not overlap. Alternatively, these two distinct sets of differences might mediate similar changes (i.e. causing the instability of Pxr transcripts).

We calculated RNA structural stabilities with the self-folding free energy ΔG to explore the possibility that the sequences of *pxr*_*Cb.1*_ and *pxr*_*Cb.3*_ in *Cystobacter* affect the stability of their respective transcripts. None of the predicted structures from species other than *M. xanthus* (ΔG values from −58.9 to −52.2, with lower values reflecting greater stability) appear to be considerably less stable than the *M. xanthus* structure (ΔG_GJV1_ = −58.3). Therefore, it is unlikely that the reduced expression of Pxr_Cb.1_ and Pxr_Cb.3_ sRNAs in *M. xanthus* is caused by differences in intrinsic structural stabilities alone. Nevertheless, the re-computed free energies of Pxr_Cb.1_ and Pxr_Cb.3_ given the consensus structure are predicted to be substantially less stable (ΔG_Cb.1_ = −38.9 and ΔG_Cb.3_ = −15.2) than Pxr_Cbm6.2_, Pxr_Cbvi34.2_ and Pxr_GJV1_ (ΔG_Cbm6.2_ = −51.4, ΔG_Cbvi34.2_ = −50.5 and ΔG_GJV1_ = −57.9), indicating that the base changes in *pxr*_*Cb.1*_ and *pxr*_*Cb.3*_ destabilize the consensus structure shared by single-copy Pxr orthologs.

## Discussion

**Fig. 5:**
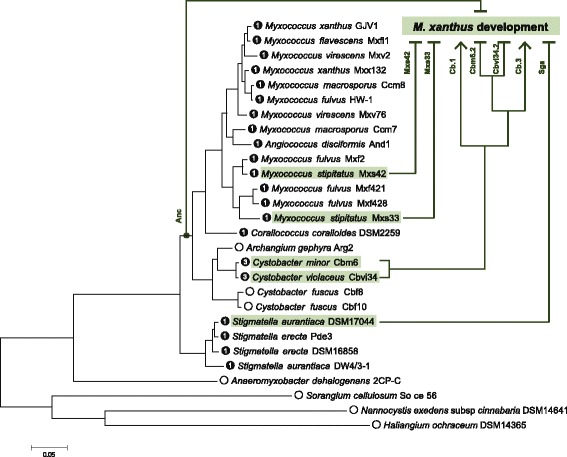
Phylogeny of myxobacterial species and strains summarizing the effects of diverse *pxr* alleles on *M. xanthus* development. The phylogenetic analysis was based on five conserved loci: 16S rRNA, 23S rRNA, *pyrG*, *rpoB* and *pgm*. Solid circles indicate that Pxr was detected in the respective species and the numbers inside indicate the numbers of *pxr* alleles found. Empty circles denote that Pxr was not detected. ‘Anc’ indicates the phylogenetic position of the ancestral *pxr* allele examined in this study. *Shaded boxes* highlight extant species from which respective *pxr* alleles were transferred into *M. xanthus* (see text for details). Lines towards the “*M. xanthus* development” box with *bar heads* and *arrow heads* indicate *pxr* alleles able vs. unable to control *M. xanthus* development, respectively. Modified from [38]

Bacterial sRNAs are a class of regulatory elements that has great importance in the function and evolution of bacterial genomes, but how their functions evolve over time remains poorly understood. Here, we tested for divergence of the bacterial sRNA Pxr in the fruiting myxobacteria by introducing sequences of its inferred ancestor and heterospecific homologs into an *M. xanthus pxr* deletion mutant. We showed that both the inferred ancestor and homologs from species with only one copy of *pxr* restored the regulatory function of Pxr observed in *M. xanthus* (Figs. 3-4), suggesting that such function has been established since its likely origin within the myxobacteria and has been conserved in different species (Fig. 5). Nevertheless, multiple paralogs present in the genus *Cystobacter* exhibited differences in their functional effects, with two paralogs controlling *M. xanthus* development but two others failing to do so (Figs. 4-5). These differences in functional effects may be due to differences in sRNA accumulation levels in turn caused by structural differences in the Pxr variants encoded by these paralogs. While previous work has examined functional differentiation of paralogous sRNAs in *Vibrio* spp. (e.g. [48–50]), here we have compared the functional effects of both orthologous and paralogous bacterial sRNAs in a common genetic background.

Our results also shed light on the sequence regions important for the function of Pxr. The *pxr* alleles we examined contain a specific set of polymorphic sites that allow us to infer their functional relevance. For the *pxr* ancestor and homologs that control development in *M. xanthus*, their sequences are highly identical in the first and part of the second stem-loops, illustrating the significance of these regions for the function of Pxr.

It has long been recognized by evolutionary biologists that genes from the same species coevolve together and during this process may become incompatible with genes from other species that are evolving independently due to reproductive isolation [51, 52]. Hybrid dysfunction caused by such independent divergence is well known in animals and plants [53]. Similarly, sRNAs from one species may not be able to control target mRNAs from another species, and this idea has been tested with the *miR-310* miRNA family across different *Drosophila* species in animals [54]. For the sRNA Pxr in the myxobacteria, the homologs we introduced into *M. xanthus* are from species diversified in fruiting body size, shape and color [43]. However, many of their Pxr sRNAs controlled *M. xanthus* development in a manner similar to the native *M. xanthus* allele, revealing strong selective constraints on the regulatory interactions of Pxr (including both processing and interactions with target mRNAs) across different species. Our results thus suggest that Pxr targets are shared by these different species and will facilitate future identification of target genes. Nevertheless, our results did not exclude the possibility that regulons of Pxr have expanded in species other than *M. xanthus*, or the possibility that Pxr may evolve to regulate additional developmental processes in other species.

We also showed that the specificity of Pxr function appears to be evolving among some of its paralogs, as the sRNAs transcribed from *pxr*_Cb.1_ and *pxr*_Cb.3_ are expressed in much lower amounts in *M. xanthus* than are transcripts from the native *M. xanthus* allele and other homologs. Most likely, the structural differences caused by sequences in these two paralogs affect the stability of the sRNA molecules transcribed from them such that low accumulation of Pxr fails to effectively block development. In our experimental design, all *Cystobacter* paralogs are transcribed from the same *M. xanthus* enhancer binding site and promoter to eliminate potential confounding effects of any variation in the effects of these regulatory elements on transcription. Because all *Cystobacter pxr* paralogs have their own σ^54^ promoters, sequence differences in the enhancer binding sites and/or in the regulatory regions upstream of the promoters may mediate differential expression and enable higher expression of *pxr*_Cb.1_ and *pxr*_Cb.3_ in their native genomic background.

It is possible that Pxr interacts with accessory proteins that modulate their expression or stability. For example, the paralogous sequences or structures in *Cystobacter* might prevent or reduce processing of Pxr-L into Pxr-S by the relevant *M. xanthus* ribonuclease and thus prevent accumulation of the active form of Pxr. Nevertheless, our results show that even the Pxr-L forms from the *Cystobacter* alleles were present in much lower amounts. In *E. coli*, the RNA chaperone Hfq protects sRNAs from degradation by ribonucleases [33]. Nevertheless, Hfq preferentially binds to AU-rich sequences [55, 56] and no homologs of Hfq were detected in the sequenced myxobacterial genomes that are generally GC-rich, suggesting that an alternative protein might serve as RNA chaperone for Pxr sRNA. It is also known that in *E. coli,* polynucleotide phosphorylase (PNPase) increases the stability of sRNAs [57]. Such accessory proteins might identify specific sequence regions or structures of Pxr in order to regulate their expression or stability, and may have coevolved with Pxr closely in addition to target mRNAs. Identification of these proteins will allow testing for adverse interactions between Pxr sRNAs and accessory proteins from different species (e.g. *Cystobacter* species), and whether such interactions result in reduced accumulation levels of Pxr sRNAs we observed in this study. Lastly, it remains unknown whether these paralogous sRNAs effectively hybridize with respective target mRNA molecules in *M. xanthus* due to instability.

Known bacterial sRNA paralogs include OmrA and OmrB responsible for high osmolarity in *E. coli* [58], PrrF1 and PrrF2 for iron limitation in *Pseudomonas aeruginosa* [39], four copies of Qrr for quorum sensing in *Vibrio cholerae* [59] and five copies in the bioluminescent marine *V. harveyi* [50] (for detailed review, see [60]). Interestingly, while the Qrr sRNAs in *V. cholerae* act redundantly, in *V. harveyi* they act additively to control the quorum sensing master regulator [50, 59], and some of the sRNAs have evolved preferences for additional targets [48, 49]. Our findings here add another example of divergence among bacterial sRNA paralogs, and lay the groundwork to study how developmental programs are regulated by sRNAs in myxobacterial species. Future work of interest includes eludication of the effects of each *pxr* paralog in their own host species in the *Cystobacter* subclade.

## Conclusions

In animals and plants, the discovery of RNA silencing led to the understanding that small non-coding RNAs are commonly used by eukaryotes to post-transcriptionally regulate the expression of many genes. In bacteria, a rapidly increasing number of small RNAs have also been found to regulate a wide variety of important biological processes. However, in most systems the functional evolution of sRNAs and how they coevolve with their associated regulatory networks remain poorly understood. Pxr is a small RNA that regulates the initiation of fruiting body development in the model myxobacterial species *M. xanthus* and appears to have a recent origin in a subclade of the myxobacteria, thus serving as a great system to study the divergence of a bacterial sRNA. We show that the regulatory function of Pxr is conserved across different myxobacterial species, generating insights into identifying functional elements and the sequence-function relations of bacterial sRNAs. Further, its function appears to have changed in some lineages through gene duplication and subsequent diversification. This provides opportunities to study the roles of sRNAs in developmental programs of the myxobacteria, which exhibit among the most complex multicellular behaviors found in bacteria.

## Methods

### Plasmid construction

All plasmids constructed in this study are listed in Table 1. One positive and one negative control plasmids (pPxr_GJV1_ and pPxr_null_, respectively) were first constructed. To construct the positive control pPxr_GJV1_ that bears the GJV1 *pxr* allele, a 537-bp PCR fragment containing the 3′-terminal 167-nt of *nla19*, the 262-nt intergenic region between *nla19* and *pxr* that contains the predicted σ^54^ promoter, and the 108-nt *pxr* coding region in *M. xanthus* was cloned into pCR2.1 vector that carries a kanamycin-resistance marker gene. To construct the negative control pPxr_null_ that carries only the 429-nt region upstream of the *pxr* gene, a PCR fragment containing only the 3′-terminal 167-nt of *nla19* and the 262-nt intergenic region between *nla19* and *pxr* was cloned into pCR2.1. Primers used for PCR amplification are listed in Additional file 1: Table S1. Competent cells of *Escherichia coli* TOP10 were used for plasmid cloning and were grown at 37 °C in Luria-Bertani (LB) medium [61] or on X-Gal/IPTG LB hard (1.5%) agar plates to screen for colonies with PCR fragment inserts. The sequences and orientation of the inserts were confirmed by sequencing.

#### The inferred *pxr* ancestor

To reconstruct the previously inferred *pxr* ancestor [38] in the laboratory, we used the genomic DNA of *M. xanthus* GJV1 as template and designed three PCR primers that contain the ancestral nucleotides in the *pxr* ancestor in order to replace the *M. xanthus*-specific sequence. Two of the primers (Node38_Bulge_For and Node38_Bulge_Rev) are the sense and anti-sense strand sequences, respectively, at the ancestral *pxr* positions 13–33 and contain the ancestral U on the bulge on the left side of the first stem-loop (Fig. 1a, b). The third primer, Node38_LongRev, is a 64-nt long primer at the positions 48–111 that contains the remaining six ancestral sites from the third stem-loop to the end of the *pxr* coding region. We first PCR-amplified a 462-bp DNA fragment that contains the 429-bp fragment preceding *pxr* in conjunction with the nucleotide positions 1–33 in the ancestral *pxr* with the primers GV367 and Node38_Bulge_Rev (5′ GGG GGG AAC C**A**C CTT CAG CCT 3′) (the bold letter indicates the ancestral nucleotide). Next, we PCR-amplified a 99-bp fragment from the positions 13 to 111 in the *pxr* ancestor using primers Node38_Bulge_For (5′ AGG CTG AAG G**T**G GTT CCC CCC 3′) and Node38_LongRev (5′ AAA AGA AGG CGG CCC GA**T** ACC CCA A**G**A GA**G** GGT **A**CC GGG CCG CGG GT**T** CTT CT**A** AAG GTG ACT C 5′). We subsequently performed a PCR to join the 462-bp and 99-bp fragments together by the overlapping sequences at the positions 13–33 in the *pxr* ancestor and to amplify the joint 540-nt fragment with GV367 and Node38_LongRev. The resulting PCR fragments were cloned into pCR2.1 and verified by sequencing.

#### Homologs of *pxr* from different myxobacterial species

To introduce different *pxr* homologs into *M. xanthus*, we first PCR-amplified the 429-bp DNA fragment preceding *pxr* coding region in *M. xanthus*. We then PCR-amplified each *pxr* homolog using the genomic DNA extracted from their host strains with primer sequences listed in Additional file 1: Table S1. Next, we ligated the 429-bp PCR products with each *pxr* homolog using T4 DNA ligase (Thermo Scientific). A subsequent PCR was performed to amplify the ligated fragment using the forward primer complementary to the 5′ region of the 429-bp fragment and the reverse primer complementary to the 3′ region of each *pxr* homolog. The resulting PCR products were cloned into pCR2.1 and confirmed by sequencing.

### *M. xanthus* strain construction

All *M. xanthus* strains used in this study are listed in Table 1. All constructed strains were derived from the strain OC Δ*pxr* and generated with plasmid integration, which was achieved by electroporation of OC Δ*pxr* competent cells with plasmids that carry the 429-nt homologous region with the predicted σ^54^ promoter preceding *pxr* in conjunction with different versions of *pxr*. To perform electroporation, overnight cultures of OC Δ*pxr* grown in CTT liquid medium [62] were harvested at exponential-growth phase by centrifugation at room temperature. The cells were washed five times with and resuspended in double-distilled water. Seventy-five microliters of the cell suspension and 3 μl of the plasmid DNA were mixed and transferred to electroporation cuvette. Electroporation was performed with a Bio-Rad Gene Pulser apparatus set at 400 Ω, 25 μF and 0.65 kV. The cells were then transferred to an Erlenmeyer flask containing 3 ml of CTT liquid and incubated overnight at 32 °C with constant shaking at 300 rpm. Samples were diluted into CTT soft (0.5%) agar containing 40 μg kanamycin/ml and incubated at 32 °C with 90% rH (relative humidity) for a week until colonies became visible. For each constructed strain, two to three clones were isolated. The genetic organization of the resulting strains is illustrated in Fig. 2.

### Developmental assay

*M. xanthus* strains were grown in CTT liquid at 32 °C with constant shaking at 300 rpm. To perform the developmental assays, cultures in exponential-growth phase were centrifuged at room temperature and resuspended in TPM liquid [63] to a density of 5 × 10^9^ cells per ml. For each strain, 50 μl of resuspended cells were spotted on the center of nutrient free TPM hard (1.5%) agar plates and incubated at 32 °C with 90% rH. After three days, cells were harvested with a scalpel blade, transferred into 1 ml of double-distilled water and heated at 50 °C for two hours to select for heat-resistant spores. Samples were then sonicated with a microtip to disperse spores, diluted and plated into CTT soft (0.5%) agar plates. After seven days, visible colonies were counted to estimate the total number of spores produced during development. In cases where no colonies grew at our lowest dilution factor (10^−1^), a value of ten spores per replicate was entered for data analysis, providing conservatively high estimates of spore production in these cases. In any given assay, two or three clones of identical constructs were tested. The same experimental procedure was repeated at least three times for all strains.

### Expression pattern and stability of Pxr sRNA

We detected the expression of Pxr sRNA by Northern blotting. The total enriched small-sized RNA preparation of each strain was extracted from vegetative cultures growing in CTT liquid with the mirVana miRNA isolation kit (Ambion). RNA concentration was measured with a Nanodrop spectrophotometer and equal amounts of RNA (1 μg) were electrophoresed in 10% SequaGel (National Diagnostics) and electro-transferred onto a BrightStar®-Plus positively charged nylon membrane (Ambion). After UV cross-linking, the membrane was pre-hybridized in 4 ml UltraHyb-Oligo buffer for 30 min and subsequently hybridized in the same solution containing 100 pmol 3’Biotin-TEG-*pxr* oligo probe (5′-ACC GGA AGT GCT GAA GGG GTG GGG GG-3′) (Sigma) overnight. Pxr sRNA was detected with BrightStar® Biodetect non-isotopic kit (Ambion). We estimated the stabilities of the Pxr ancestor and homologs in one stem-loop structure by computing the self-folding free energy ΔG of their RNA sequences using Mfold [64]. The consensus secondary structure of Pxr based on the alignment of single-copy *pxr* homologs was predicted with RNAalifold within the ViennaRNA software package [65]. We re-estimated the ΔG of the homologs given the consensus structure with the RNAeval function [66].

## Additional file

Additional file 1: Table S1. Primers used in this study. (DOCX 17 kb)

## Abbreviations

miRNAs: microRNAs
Pxr-L: the long form of Pxr sRNA
Pxr-S: the short form of Pxr sRNA
sRNAs: small non-coding RNAs
