# Supplementary material for: Divergence of functional effects among bacterial sRNA paralogs
Source: BMC Evol Biol. 2017 Aug 22;17:199. doi: 10.1186/s12862-017-1037-5 (PMC5568312; doi:10.1186/s12862-017-1037-5)
Supplement: Additional file 1: Table S1. — Primers used in this study. (DOCX 17 kb) [file 12862_2017_1037_MOESM1_ESM.docx]

**Table S1.** Primer Sequences

| Amplified fragment | Forward primer sequence | Reverse primer sequence |
| --- | --- | --- |
| *pxr*_GJV1_ | CCC AGG TGG TGG AAG AGG | CGC AGC ACC CAC TGA GAT TC |
| *pxr*_null_ | CCC AGG TGG TGG AAG AGG | CCT TTC GTC GCG AGC CGA TGT GTC CCG CGC ATT CC |
| 462-bp fragment^a^ | CCC AGG TGG TGG AAG AGG | GGG GGG AAC C**A**C CTT CAG CCT |
| 99-bp fragment^a^ | AGG CTG AAG G**T**G GTT CCC CCC | AAA AGA AGG CGG CCC GA**T** ACC CCA A**G**A GA**G** GGT **A**CC GGG CCG CGG GT**T** CTT CT**A** AAG GTG ACT C |
| 429-bp fragment preceding *pxr* | CCC AGG TGG TGG AAG AGG | CGA TGT GTC CCG CGC ATT CCT A |
| *pxr_Mxs33_* | AAG CGA GGC TGA AGG CGG TT | ATG CCC AAA AGA AGG CGG CCC GAC AC |
| *pxr_Mxs42_* | AAG CGA GGC TGA AGG CGG TT | CAG CAC AAA AGA AGG CGG CCC GAC AC |
| *pxr_Cb.1_* | ACG TGG CGA TGA AGG TGG TTC CCC CCC ACC C | GGC CCA AAA AGA AGG CGG CCC GGT CCC CTC AGG |
| *pxr_Cbm6.2_* | AAG TGA GGC TGA AGG AGG TTC CCC CCC ACC G | GGC GCA AAA AGA AGG AGG CCC GGT CAC CTC AGA |
| *pxr_Cbvi34.2_* | AAG TGA GGC TGA AGG AGG TTC CCC CCC CAC CG | GGC GCA AAA AGA AGG AGG CCC GGT CAC CTC AGA |
| *pxr_Cb.3_* | AAG TGA GGC TGA AGA AGG TTC CCC CCC ACC T | GCC CAA AAA AGA AGG CGG CCC GGC ATC CTC AGG |
| *pxr_Sga_* | AAG CGA GGC TGA AGG TGG TT | GGG CTA GAA AAG AAG GCG GCC CGA TA |

^a^ For the reconstruction of the inferred *pxr* ancestor. The bold letters indicate the ancestral nucleotides.
